# ‘This won’t hurt a bit!’ – A descriptive review of health care professionals’ pharmacological management of pain in minor trauma

**DOI:** 10.4102/safp.v63i1.5249

**Published:** 2021-04-22

**Authors:** Duncan M. Havenga, Jaykumaran Govender, Carolyn Lewis

**Affiliations:** 1 Division of Emergency Medicine, Nelson Rolihlahla Mandela School of Medicine, University of KwaZulu-Natal, Durban, South Africa; 2 Division of Emergency Medicine, Faculty of Health Sciences, University of the Witwatersrand, Johannesburg, South Africa

**Keywords:** analgesia, trauma, emergency centre, developing countries, rural medicine

## Abstract

**Background:**

Emergency Centres (ECs) have a prominent trauma burden requiring effective pain management. This study aimed to review analgesia-prescribing habits in minor trauma, reviewing the patient demographics and diagnoses, analgesia-prescribing habits of health care professionals (HCPs) managing these cases, and differences in prescribing noted by patients’ age group, gender and triage code.

**Methods:**

A prospective, cross-sectional, descriptive study was conducted in a regional EC in KwaZulu-Natal. HCPs managing minor trauma patients completed a closed-ended questionnaire which indicated the patients’ demographics, diagnosis and analgesia prescribed.

**Results:**

The study comprised of 314 cases of which the demographic most represented were male patients aged between 20–30 years with soft tissue injuries. Simple analgesics and weak opioids (paracetamol, ibuprofen and tramadol) accounted for 87.9% of prescriptions. Referral clinics prescribed less analgesics than that provided in the EC. There were mostly no significant differences in prescription habits by patients’ age group, gender and triage code.

**Conclusion:**

Presenting complaints in our study were varied and likely to result in mild to moderate pain. Only a minority of patients received analgesics at initial contact. Standardised protocols providing treatment guidance for nurse-initiated pain management at initial contact is thus important. There were no significant differences in analgesics prescribed for adults and the elderly, which is worrisome given the potential negative side effects of analgesics in the elderly. Similar concerns in our paediatric population were not noted. Ensuring adequate analgesia with cognisance for safety at the extremes of age is of paramount importance.

## Introduction

Emergency Centres (ECs) in African regions such as KwaZulu-Natal (KZN) face many challenges, including the high burden of trauma cases that are seen on a daily basis. The World Health Organisation (WHO) estimates that the global mortality rate from injuries is around 5 million per annum.^1^ In Africa, road accidents and interpersonal violence are highly prevalent and are on the increase over the last 10 years.^2^ There is a paucity of data reporting on minor cases of trauma that constitute a large portion of the workload. Trauma-related injuries contribute around 25% of the workload in most state hospital ECs in KZN.^3^ Pain, initiated by inflammatory mediators, accompanies most tissue injury. Hence, whilst not all trauma is necessarily painful, given that trauma has the potential to cause pain, effective pain management is an essential skill for any healthcare professional (HCP) working in an EC.

Analgesia-prescribing habits vary amongst HCPs in ECs around the world.^4^ Initial analgesia prescribing habits amongst HCPs working in ECs have been found to be notoriously poor for pain management, potentially because of under assessing patients’ pain.^5^ Prescribing habits can be substantially improved with pain management seminars and nurse-driven triage protocols for pain assessment and management.^6,7,8^ The concept of oligo-analgesia (single drug administration in the treatment of pain) is a concern in ECs as this may result in inadequate analgesia and hence, protocols for stepwise increments of pain management should be implemented.^9^ A standardised pain management protocol can greatly improve a patient’s hospital experience and overall satisfaction.^10^

The aim of this study was to review analgesia prescribing habits for minor trauma in the EC. To achieve this, the study reviewed the demographics of patients presenting with minor trauma; assessed the types of minor trauma presentations to the EC; assessed what analgesics were prescribed when HCPs faced minor trauma cases in a state hospital’s EC; described the contact point within the healthcare system when the analgesia was prescribed as well as the route of administration, and described any differences in types of analgesics or dosages noted by patients’ age group, gender and triage code.

## Methods

A prospective, cross-sectional, descriptive study was conducted in the EC of Edendale Hospital, a 900-bed regional state hospital in KZN which serves a mix of rural and urban patients. The study was conducted over a period of 6 months from 01 May 2019 to 31 October 2019. It included all minor trauma patients – coded green or yellow, according to the South African Triage Scale (SATS).^11,12,13^ The SATS uses physiological parameters, symptoms and certain modifiers, to categorise patients into triage categories: green, yellow, orange, red and blue. Patients categorised as green or yellow codes were considered to be minor trauma patients for this study.

The following HCPs were based in the EC at the study site during the study period: four emergency medicine specialists (consultants), five emergency medicine registrars, seventeen medical officers, three clinical associates and five medical students. The medical students’ prescriptions were subsequently supervised and co-signed by a doctor. Thus, the EC is staffed by a varied range of HCPs with differing levels of expertise and experience. Written consent was completed by each HCP prior to participation. All HCPs working in the EC were asked to complete data collection sheets voluntarily and anonymously in the form of a survey with a closed-ended questionnaire for patients that they treated during their shift that had presented with minor trauma, over the study period. All ranks of HCPs working in the department were represented in the data collection sheets that were completed. It was anticipated that not all HCPs would complete data collection sheets, and data collection sheets would not be completed for all patients seen. Thus, a time frame of 6 months was chosen in order to collect data collection sheets from a wide variety of HCPs for a wide range of presenting pathologies in order to gain a representative, albeit convenience sample.

Demographic data collected included the patients’ age, gender, diagnosis and triage code. A list of commonly prescribed analgesics was listed in the survey with a tick box of both which analgesic the patient received and when the patient received the medication from the registered or professional nurse at the referring clinic (if applicable), on arrival at the EC as part of a triage-initiated analgesia programme (on presentation), during treatment in the EC, or dispensed on discharge. Space was available to add other medications given that were not listed on the data collection tool. The study neither addressed non-pharmacological strategies for pain management, nor did it address pain management by means of nerve blocks.

For each data collection sheet completed, space was provided for the HCP in the EC to document analgesia prescribed and administered at a clinic level. Referral letters from surrounding clinics were assessed by the attending HCP after completing the data collection sheet to determine if analgesia was administered by the HCP (registered or professional nurses) working in the referral clinics. Thus, analgesia given at that point in the continuum of care was captured.

Data collection sheets were placed in a sealed box in the EC upon completion by the HCPs. These data collection sheets were collected periodically, and the data collected was entered by the author onto a password protected Microsoft Excel (Microsoft Excel [computer program] version 14.0.4734.1000, Microsoft Corporation, 2010) document. The data was cross-checked by the corresponding author by single person double entry.

The information collected was analysed using Stata (Stata Statistical Software [computer program]. Release 15. StataCorp. College Station, TX: StataCorp LLC; 2017.) to create descriptive statistics such as frequencies and percentages to summarise patient demographics, triage codes and to classify types of minor trauma presentations. The patients’ age distribution did not follow a normal distribution; it was analysed using a visual evidence histogram and the Shapiro-Wilk test, and therefore the median (with interquartile range) was reported to analyse the distribution.

Frequency tables were constructed to summarise and analyse medication prescribed by referral clinics, to analyse which medications were prescribed at each point within the continuum of care and to analyse medications prescribed, both by type of analgesia and dosages for age groups of patients (children 1–17 years of age; adults 18–60 years of age and elderly > 60 years of age), patients’ gender and SATS code.

The Pearson chi-squared test was used to analyse if significance existed between the type of medication prescribed by age group, gender and SATS code. The Kruskal Wallis test was used to assess if a significant difference existed between dosages prescribed between age groups. The Mann-Whitney U test was used to describe if any significant differences existed between dosages prescribed and patients’ gender and SATS code.

### Ethical considerations

Ethical approval was granted by the University of KwaZulu-Natal Biostatistics Research Ethics Committee (BREC reference: BE664/18) and KwaZulu-Natal Department of Health (KZ_201902_028).

## Results

**FIGURE 1:**
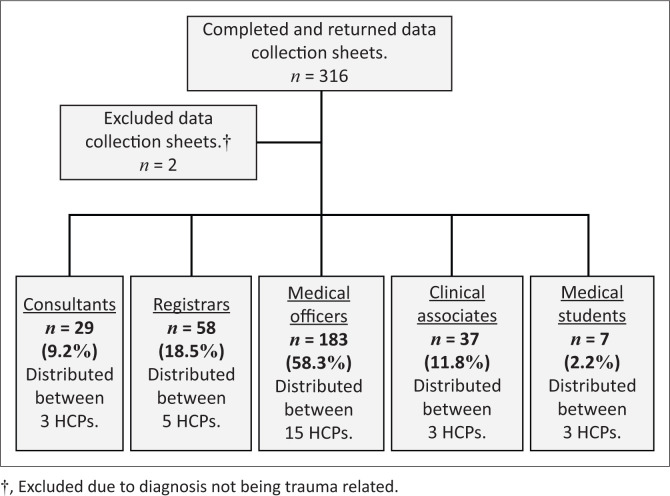
Breakdown of completed data collection sheets by healthcare professionals.

The study focused on the analgesics prescribed in patients presenting with minor trauma within the EC of a regional state hospital in KZN. All HCPs working in the EC at the study site were invited to complete data collection sheets for all patients attended to with minor trauma over the study period. A total of 316 data collection sheets were collected over the 6-month study period of which 314 were included in our study. Completed data collection sheets were represented by all ranks of HCPs (Figure 1).

### Patient demographics

**FIGURE 2:**
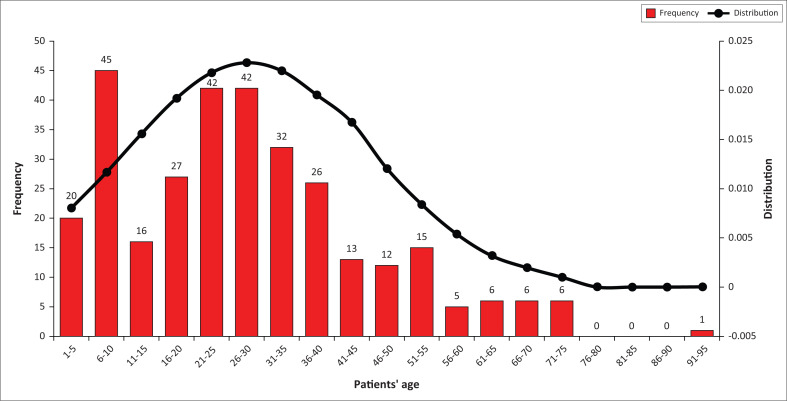
Frequency and distribution of patients’ age.

Most patients included in the study were male patients (*n* = 192; 61.1%). The age distribution of patients presenting with minor trauma-related injuries was mostly patients 40-years old or younger (*n* = 250; 79.6%) making up close to 80% of the study population (Figure 2), with the highest number of patients presenting with minor trauma injuries in the third decade of life (*n* = 84; 26.8%). Their median age was 26.5 years (interquartile range of 14–37). The minimum and maximum ages were 1 and 93, respectively.

Of the 314 patients seen, the SATS indicated that 250 (79.6%) were triaged green and 64 (20.4%) were triaged yellow. Eighty-five (27.1%) were children and 229 (72.9%) were adults. According to SATS^11,12,13^ open fractures are triaged as orange and closed fractures are triaged as yellow (modifiers). However in our study, the data collection sheets reflected that a large portion of fractures were triaged green (Table 1). This may have been in error by the triage process (not taking into consideration the modifier).

**TABLE 1:** Patient diagnoses, triage score and age distribution.

Patient Diagnosis	SATS Score	SATS Score	Total	
	Green	Yellow	*n*	%
**Soft tissue injury**	-	-	121	38.5
Adult	73	14	-	-
Child	23	11	-	-
**Laceration**	-	-	48	15.3
Adult	25	8	-	-
Child	14	1	-	-
**Fracture**	-	-	48	15.3
Adult	19	8	-	-
Child	17	4	-	-
**Motor Vehicle Accident**	-	-	22	7.0
Adult	13	2	-	-
Child	7	0	-	-
**Penetrating Knife Wounds**	-	-	20	6.4
Adult	18	2	-	-
Child	0	0	-	-
**Assault**	-	-	13	4.1
Adult	6	7	-	-
Child	0	0	-	-
**Miscellaneous**	-	-	12	3.8
Adult	7	2	-	-
Child	3	0	-	-
**Pedestrian Vehicle Accident**	-	-	12	3.8
Adult	8	2	-	-
Child	2	0	-	-
**Falls**	-	-	7	2.2
Adult	6	0	-	-
Child	1	0	-	-
**Head Injury**	-	-	6	1.9
Adult	5	1	-	-
Child	0	0	-	-
**Dog Bites**	-	-	3	1.0
Adult	1	1	-	-
Child	1	0	-	-
**Burns**	-	-	1	0.3
Adult	0	0	-	-
Child	0	1	-	-
**Gun Shot Wound**	-	-	1	0.3
Adult	1	0	-	-
Child	0	0	-	-
**Total**	-	-	314	100
Adult	182	47	-	-
Child	68	17	-	-

Child: Age < 18 years old; Adult: Age ≥ 18 Years old.

SATS, South African Triage Scale.

The most diagnosed minor trauma-related injury (Table 1) was soft tissue injuries (*n* = 121; 38.5%). The term soft tissue injury is used to describe injuries to the soft tissues in the body, rather than the harder bones: for example bruises, sprains and muscle contusions.^14^

### Analgesia prescribed

Analgesia was prescribed by the HCPs (registered or professional nurses) working in referral clinics prior to arrival in the EC in 45 cases, making up a total of 59 medications prescribed (Table 2) out of the 976 total medications prescribed, with an average of 1.3 medications prescribed per patient encounter at clinic level. A total of 917 medications were prescribed within the EC representing an average of 2.9 medications prescribed per patient encounter within the EC. This suggests that oligo-analgesia occurs predominantly at clinic level with a more multimodal approach to pain management within the EC.

**TABLE 2:** Analgesia prescribed by healthcare professionals working in the referral clinics.

Drug prescribed	Total	
	*n*	%
Paracetamol	42	4.3
Ibuprofen	12	1.2
Diclofenac	3	0.3
Morphine	1	0.1
Pethidine	1	0.1

**Total**	**59**	**6.0**

The most prescribed analgesics were paracetamol (48.0% of all prescriptions), ibuprofen (24.9% of all prescriptions) and tramadol (9.4% of all prescriptions) and this was representative throughout all the contact points within the continuum of care within the EC (Table 3). Paracetamol and ibuprofen were prescribed in combination multiple times by the clinic (12), on presentation (24), during treatment (55) and on discharge (237) by HCPs.

**TABLE 3:** Prescribed analgesia within the continuum of care in the EC.

Medication	Presentation		Treatment		Discharge		Total	
	*n*	%	*n*	%	*n*	%	*n*	%
Paracetamol	98	10.0	116	11.9	255	26.1	469	48.0
Ibuprofen	25	2.6	58	5.9	160	16.4	243	24.9
Tramadol	9	0.9	33	3.4	50	5.1	92	9.4
Morphine (IVI/IMI)	10/1	1.0/0.1	29/0	3.0/0.0	1/0	0.1/0.0	40/1	4.1/0.1
Ketamine (IVI/IMI/PO)	0/1/0	0.0/0.1/0.0	2/9/1	0.2/0.9/0.1	0/0/0	0.0/0.0/0.0	2/10/1	0.2/1.0/0.1
Tilidine (Valoron drops)	0	0.0	8	0.8	0	0.0	8	0.8
Diclofenac IMI	1	0.1	1	0.1	1	0.1	3	0.3
Fentanyl IVI	0	0.0	3	0.3	0	0.0	3	0.3

**Total†**	**145**	**14.9**	**260**	**26.6**	**467**	**47.8**	**872**	**89.3**

Presentation: Medication prescribed on presentation to hospital; Treatment: Medication prescribed during treatment of the patient; Discharge: Medication prescribed on discharge of the patient for ongoing out-patient treatment.

PO, per os; IVI, Intravenous injection; IMI, Intramuscular injection.

† , Totals exclude prescriptions in the EC of pethidine, naproxen, indomethacin, ethyl chloride (1 prescription each) and lignocaine (only prescribed during treatment).

Lignocaine was prescribed during the treatment stage in the continuum of care only when suturing or wound closure was undertaken. Ketamine was prescribed as an intramuscular injection a total of nine times during the treatment stage in the continuum of care – all to paediatric patients – for procedural sedation for either wound closure or burns management. Pethidine, naproxen and indomethacin were prescribed on discharge, each for a single patient only, all for soft tissue injuries. Ethyl chloride spray was prescribed and used once during treatment for management of a soft tissue injury to a finger, with delayed presentation resulting in a paronychia.

There were no statistically significant differences when comparing which analgesic medications were prescribed by age group (paediatrics/children 1–17 years of age; adults 18–60 years of age and elderly > 60 years of age). Of note, non-steroidal anti-inflammatory drugs (NSAIDs) were prescribed with equal frequency in the elderly as in other age groups. Sixty-five per cent of elderly patients received ibuprofen orally, 10% of elderly patients received diclofenac IMI and 5% of elderly patients received naproxen orally. There were no statistically significant differences between dosages prescribed for the adult age group and the elderly across all medications, inclusive of potent opioid analgesics.

There were no statistically significant differences between either type of analgesic medication prescribed or the dosage of analgesic medication prescribed by gender.

Morphine IVI was prescribed more commonly for patients triaged yellow versus green by SATS triage code (27% vs. 10% *p* = 0.00), as was ketamine IMI (8% vs. 2% *p* = 0.02). There were no statistically significant differences for any other type of analgesic medications or dosages by triage code.

## Discussion

ECs in Africa and KZN have a high burden of trauma^2,3^ and thus effective pain management is an important skill for HCPs.

The demographic most affected by minor trauma are male patients between 20 and 30 years of age, of which the majority of injuries are due to soft tissue injury.^14^ This is comparable with studies undertaken in other African countries^15,16^ as well as within South Africa assessing both major trauma^17^ and all trauma^18,19^ which noted a similar demographic representation. Our study suggests that although soft tissue injuries predominated in our data sample, minor trauma is a very diverse category of presentation likely to result in mild to moderate pain.

Paracetamol, ibuprofen and tramadol were the most prescribed analgesics, accounting for 87.9% of the medication prescribed.

Paracetamol accounted for more than half of all analgesic prescriptions. These trends are comparable with several other studies worldwide inclusive of low- and middle-income countries,^20,21,22,23^ and can likely be attributed to the safety profile of paracetamol. Differing studies, again from both high- and middle-income as well as low-income countries have, however, reported that NSAIDs such as ibuprofen are the most commonly prescribed analgesic in minor trauma.^24,25^ Although our study notes that paracetamol is the most prescribed analgesic, we do note a high trend in the prescription of ibuprofen. Notably, most patients who received ibuprofen, within in the continuum of care, received it as a combination with paracetamol.

Tramadol is a weak opioid and was the most prescribed opioid in our study. It is likely that this is because of a combination of its favourable side effects profile resulting in less respiratory depression, dependency and constipation, compared to other opioids as well as ease of availability.^26^ A study in Nigeria noted that codeine is the most commonly prescribed opioid for pain,^20^ however codeine is not widely available in the state ECs in KZN and this may account for tramadol being the most commonly prescribed opioid as opposed to codeine in our study. In fact, codeine is not represented in our results at all, most likely as a result of its lack of availability in the state ECs. Limitations in availability of analgesics, such as codeine, indomethacin and ketorolac in the state ECs, as well as the lack of familiarity of HCPs working in the EC with certain analgesics, such as diclofenac, naproxen, mefenamic acid, carbamazepine and amitriptyline, will have contributed to the high prescribing trends of paracetamol, ibuprofen and tramadol in this study.

Potent analgesics such as morphine, fentanyl, tilidine and ketamine were prescribed less frequently. This is possibly representative of the population studied in our survey, focussing on minor trauma and therefore, patients’ requirement in terms of pain control was likely achieved with less potent analgesics. It is however, a limitation of the study that the patients’ satisfaction with regards to treatment was not objectively reviewed at each point in the continuum of care. It would have been beneficial to assess if escalation or de-escalation of pain management mirrored requirements following a response to treatment.

Our study reveals that many patients were treated with non-opioid analgesics, with paracetamol and ibuprofen making up 78.5% of the total analgesics prescribed. Fewer patients were treated with a weak opioid such as tramadol (9.4%), and even fewer were treated with a strong opioid such as morphine and fentanyl (4.2% and 0.3% respectively). More potent analgesics such as morphine IVI tended to be prescribed for patients with a higher triage code which could be considered appropriate, as these patients are likely to have more severe injuries and thus potentially more severe pain. However, to regard this as appropriate with certainty, regular assessment of the patients’ response to pain would be necessary. The WHO describes a stepwise approach to pain management.^27^ A fundamental aspect of the WHO’s stepwise approach to pain management is regular assessment and reassessment of pain, and the response to treatment. Our study unfortunately did not establish where on the WHO pain ladder the patients’ initial analgesic requirement was and it did not reassess a response to the analgesia provided. Therefore, it is unknown from this study whether adequate implementation of the WHO’s stepwise approach to analgesia was achieved by HCPs working within the EC.

Underreporting could have occurred within the study for certain analgesics. Forty-eight patients had a diagnosis of laceration, but subcutaneous lignocaine use was only documented on 41 occasions. Alternatively, it is possible that lignocaine may not have been used in the management/suturing of lacerations as they were superficial and did not require closure or because of the practice of procedural sedation, which is growing in popularity within ECs in Africa.^28^ Ketamine IMI was administered on four occasions as procedural sedation, all to paediatric patients in situations where lignocaine was not used.

Given that there has been an increase in the volume of patients making use of primary healthcare facilities within the public sector in South Africa, particularly amongst those living in rural areas,^29^ it is imperative that adequate analgesia is provided for patients at the clinic level. Our study assessed what analgesics were prescribed at the clinic level by registered and staff nurses. Of the 45 patients that did receive analgesia at the clinic level an average prescription rate of 1.3 medications per patient was prescribed. Without an assessment of patients’ satisfaction with regard to treatment of pain, it is impossible to ascertain whether this relative oligo-analgesia resulted in inadequate pain relief, or whether analgesia requirements were adequately met. It is important that there is a focus on strengthening the district platform within South Africa with regards to training on assessment of pain and early provision of adequate analgesia, as this will inevitably result in better patient management at the clinic level.^6,7,8^

On arrival at the EC, patients are triaged according to the SATS.^11,12,13^ The SATS provides guidance on the time frame within which patients should be seen by an attending doctor in ECs by triage code. It is recommended that patients triaged ‘yellow’ be seen within 1 h and patient’s triaged ‘green’ be seen within 4 h. Despite these recommendations it is our anecdotal experience that because of patient overcrowding, which is a frequent problem in our ECs, patients may wait in excess of these time frames to be seen.^30,31^ Thus, there has been much discussion in the literature of the advantages of nurse-driven analgesia at triage.^7,8^ Our study has demonstrated that of a total of 976 individual medications prescribed for patients with minor trauma, only 145 (14.9%) were prescribed at presentation (at triage). Given that the next point in the continuum of care that the patient may be assessed may be several hours post triage, it is possible that analgesia requirements are not being met timeously. The introduction and/or augmentation of nurse-initiated triage of pain and protocol-driven management of pain upon presentation of the patient to the EC could potentially improve both the patient’s experience within the EC and wound healing itself. Inflammatory mediators released in response to tissue injury typically contribute to healing through their vasodilatory and pro-angiogenic stimulation of healing. However, pain that is poorly controlled, especially chronic pain, may be associated with an imbalance of mediators that result in protracted inflammation and delayed wound healing with neuropathic pain.^32^ The majority of analgesics were prescribed by HCPs during treatment or upon discharge.

It is interesting to note that there is no statistically significant difference in the types of analgesics prescribed across different age groups (children, adults and the elderly) in our study. Previous studies have suggested that pain management in paediatric patients is not prioritised by HCPs, mainly because of a lack of both knowledge and expertise amongst HCPs.^33,34^ There are a number of challenges that affect the HCPs assessment of pain in children, including language barriers (particularly in pre- or nonverbal children) and cultural differences.^33^ Again, a previous study comparing adults and paediatric patients noted that analgesics were prescribed less commonly in paediatric patients and at lower relative dosages.^34^ It is thought that this may be because of HCPs being less comfortable both, in assessing pain and providing pain relief – with particular concern about the safety aspects of prescribing certain analgesics such as narcotic analgesics.^34^ It is possible that this concern for inadequate pain management in paediatric patients is not seen in our study because of the Hawthorne effect,^35^ in that HCPs were aware that their prescribing habits were being monitored and therefore they were more vigilant in terms of what analgesia they prescribed. It may also be possible that as a result of previous educational drives, paediatric analgesic needs are appropriately assessed and attended to.

In our study there were also no statistically significant differences in both the types of analgesics prescribed and the dosages of these analgesics in the elderly patient population. Previous studies have indicated that elderly patients are at high risk of having their pain inadequately managed.^36^ Typically, this is as HCPs are cautious of treating pain in elderly patients as the elderly often do not tolerate common pain treatment advocated for adults well (such as NSAIDs or narcotic analgesics), are sensitive to drug-drug interactions and have multiple co-morbidities which may impact on the pharmacology of analgesic medications prescribed.^36^ It has been noted that polypharmacy and the indiscriminate use of NSAIDs is a major problem in the elderly.^36^ It is of possible concern that the elderly patients in our study received NSAIDs and opioids at similar rates and dosages as the adult population group. Potentially this could have resulted in unwanted side effects – an aspect that was not specifically addressed in our study – and warrants further investigation. A limitation of our study may be that because of both the low number of elderly patients represented in our study sample as well as the low number of opioids prescribed, especially potent opioids, this may have diluted the possible statistical effect.

Previous studies have shown conflicting results in terms of prescriptions of analgesics by gender. Earlier studies did appear to suggest that female patients were prescribed more analgesics and more potent analgesics than male patients.^37^ It is thought that female patients reported more pain than their male counterparts and are perceived to experience more pain by the attending HCP. The reasoning for this was postulated to be social – it is more socially acceptable for female patients to express pain and, when pain is expressed by a patient, it is more likely that HCPs will respond to a female patient.^38^ However, more recent studies seem to have contradicted this, showing no particular bias based on patients’ gender.^39^ Certainly, in our study, there was no statistically-significant difference in either the types of analgesics prescribed, or the dosages of these analgesics by gender.

There are several limitations to our study. Referral letters from clinics were used to assess what analgesics were prescribed at a clinic level. It is possible that analgesia may have been prescribed and administered at the clinic level, but not documented in the referral letter and thus analgesia prescribed at this point in the continuum of care would not have been correctly captured in our data collection. Although all HCPs working in the EC were invited to participate in the study and complete data collection sheets, it is possible that not all HCPs participated. Furthermore, data collection sheets were not completed for every case of minor trauma attended to. This convenience sampling model therefore has the potential to introduce bias into the study. In order to mitigate this, a longer sampling time frame of 6 months was chosen in an attempt to ensure that a wide range of minor trauma cases were represented and all ranks of HCPs were represented in the data collection. Consideration of the possible negative effects of the use of analgesics, such as side effects and cost analysis, were not included in this study. Furthermore, several confounding factors have been identified in this study. There is limited availability and accessibility of analgesics in both the state regional hospital’s EC and the surrounding referral clinics. Allergies of patients to medications were not accounted for. The Hawthorne effect^35^ may have made HCPs more aware of the need to prescribe adequate analgesia during the study. Data may have been affected if patients were transferred out of the EC for ongoing care elsewhere, in which case analgesics prescribed on discharge would not have been captured in our data.

## Conclusion

Patients presenting with minor trauma in our study were predominantly male patients aged between 20 and 30 years of age. The minor trauma prompting presentation for patients in our study was varies, with soft tissue injuries predominating. Given that tissue injury results in release of inflammatory mediators that often result in pain, the minor trauma presentations noted in our study are likely to have resulted in mild to moderate pain. Trauma-related pain has been shown to be best managed timeously and in a multimodal fashion.^40^ Pain control is of paramount importance in managing both the trauma patients’ experiences through an EC and, by managing pain early and appropriately, the risk of chronic pain with an imbalance of inflammatory mediators potentially resulting in delayed wound healing is mitigated.

Only a small number of patients in our study received analgesia prescribed by an HCP at clinic level. The analgesia prescribed tended to be predominantly simple analgesia such as paracetamol and ibuprofen. There is a role for nurse-initiated assessment and management of pain at the clinic level. Oligo-analgesia (which has been demonstrated in our study at the clinic level) may be inappropriate unless the patients present with mild pain that is well controlled with oligo-analgesia.

Analgesics prescribed in the EC tended to be simple analgesics and less potent opioid analgesics, which may suggest a stepwise approach to analgesia as suggested by the WHO. It is noted that only a minority of patients received analgesia at triage and thus again, there is a role for nurse-initiated assessment and management of pain on presentation to the hospitals’ ECs (at triage).

Our study has demonstrated that analgesia prescribed at extremes of age (paediatric and elderly patients) should always be given special consideration. This is to ensure that both, adequate analgesia for the patient is prescribed timeously and that the safety aspects at these extremes of age are well thought-out.

It is a major limitation of the study that patients’ experience of pain was not assessed and that likewise, response to analgesia was not assessed.

Further research taking into account patients’ assessment of pain and perceived effect of analgesia could be undertaken and could incorporate other ECs within Southern Africa to compare with the findings suggested in this study.
